# Preventive effect on endothelial surface layer damage of Fusu agent in LPS-induced acute lung injury in rats

**DOI:** 10.1007/s11010-018-3378-9

**Published:** 2018-06-14

**Authors:** Peiyang Gao, Chengshi He, Chuantao Zhang, Baixue Li, Yiling Guo, Wen Zhao, Quan Xie, Xuemei Zhang

**Affiliations:** 1 Critical Care Medicine, Affiliated Hospital of Chengdu University of Traditional Chinese Medicine, Chengdu, China; 2 Department of Respiratory, Affiliated Hospital of Chengdu University of Traditional Chinese Medicine, Chengdu, China; 3 Infectious Disease Department, Affiliated Hospital of Chengdu University of Traditional Chinese Medicine, Chengdu, China; 4 Chengdu University of Traditional Chinese Medicine, Chengdu, China; 5 Department of Emergency, Affiliated Hospital of Chengdu University of Traditional Chinese Medicine, Chengdu, China

**Keywords:** Fusu agent, Endothelial surface layer, Acute lung injury, Lipopolysaccharide (LPS), Proteoglycans

## Abstract

Acute lung injury (ALI) is one of major causes of morbidity and mortality in intensive care. In pathophysiological events of ALI, endothelial surface layer (ESL) injury can result in capillary leakage as the initial event. The “Fusu agent”, a traditional Chinese medicine, can inhibit inflammatory factors, attenuate lung capillary leak as seen in our previous study. This study was aimed to explore the molecular mechanism of Fusu agent treatment with ALI. Consistent with previous studies, we found that Fusu agent has the protective effect on LPS-induced ALI model rats. Further investigation demonstrated that heparanase activation is necessary for the LPS-induced ALI model to aggravate ESL loss. Fusu agent can inhibit heparanase activation and heparan sulfate proteoglycans’ (HSPGs) degradation to mitigate the ESL injury. Furthermore, TNF-α and intercellular adhesion molecule-1 (ICAM-1) were significantly reduced upon Fusu agent pre-treatment to inhibit inflammatory cell influx and neutrophil adhesion in ALI. These findings shed light on the pharmacologic basis for the clinical application of traditional Chinese medicine in treating ALI.

## Introduction

Acute lung injury (ALI) and acute respiratory distress syndrome (ARDS) are major causes of death in intensive care. The population-based data suggest that incidence of acute lung injury and ARDS is 1.5–8.3 per 100,000 person-years, likely to double in the next decade [1–3]. At present, therapeutic interventions of ALI or ARDS also remain limited with lacking an accepted diagnostic test and rely on a series of clinical findings for diagnosis. Search for effective and developing therapeutic drugs is still urgently needed [4].

The pathophysiologic characters of ALI are systemically complex process, including lung interstitial edema, accumulation and activity of inflammatory leukocytes, increased permeability of endothelial and epithelial barriers [5]. In pathophysiological events of ALI, vascular endothelial cell and endothelial surface layer (ESL) injury is the initial event, the final result is the alveolo-capillary barrier leakage, leading to interstitium and alveoli edema formation [6, 7]. Highly hydrated heparan sulfate proteoglycans (HSPGs) and heparan sulfate form a substantial endothelial surface layer that acts as the primary barrier to circulating inflammatory cells and large molecules in the systemic circulation. So, the ESL permeability is heavily dependent on the endothelial glycocalyx [8, 9]. HSPGs are composed of a core protein and one or more heparan sulfate (HS) chains. In these protein families, glypicans (GPC) and syndecans (SDC) contain mostly HS side chains and comprise moderately core proteins. Glypicans are linked to the cell membrane by glycosylphosphatidylinositol. Syndecans are type I transmembrane proteins with up to five glycosaminoglycans attachment sites [10, 11]. Heparanase (HPA)is an only endo-β-D-glucuronidase capable of cleaving HS side chains at a limited number of sites in vivo. Studies implicate that heparanase activity impacts on cancer metastasis, inflammation, neovascularization, and autoimmunity [12, 13]. Furthermore, the secreted heparanases can degrade basement membrane HSPGs at the sites of injury or inflammation, release factors regulating cell proliferation and angiogenesis and allow extravasation of immune cells into nonvascular spaces [14].

The development of intravital microscopy technology provides an ideal means of studying the ESL and glycocalyx structure during ALI disease. In vivo and in vitro experiments show that endotoxin/lipopolysaccharide (LPS) induces ESL loss via activation of heparanase. The potential mechanism, such as the activation of constitutively expressed endothelial heparanase, cleaves HSPGs from proteoglycan core protein to trigger the pulmonary ESL loss that occurs after LPS exposure. At the same time, endotoxin directly mediates the release of vasoactive mediators and molecules altering lung permeability; attenuates intercellular adhesion molecule-1 (ICAM-1), TNF-α, thromboxane A2 and interleukin 10 (IL-10) [15–17].

The “Fusu agent”, a traditional Chinese medicine made from *Aconitum carmichaeli Debx, Carapax Testudinis, Fructus Amomi, Rhizome Zingiberis, Radix Glycyrrhizae Preparata and Herba Ephedrae*, has been clinically used in China for the treatment of ALI. Our previous clinical and experimental studies showed that “Fusu agent” can inhibit inflammatory factors, attenuate lung capillary leak and improve the clinical effect of LPS-induced ALI, but the molecular mechanism is not clear [18, 19]. Previous study showed that LPS induces ESL loss via activation of heparanase to trigger lung capillary leak, which is the molecular mechanism hypothesis of “Fusu agent” in treating ALI in the present study.

## Materials and methods

### Experimental animals

All the SPF adult male Wistar rats (weighing 220–250 g) were from DaShuo Laboratory Animal Technology Co. Ltd, Chengdu, China. Experimental protocols were approved by Chengdu University of Traditional Chinese Medicine Affiliated Hospital Medical Ethics Committee (No.2017DL-001). All animals were housed individually in the animal center of Chengdu University of Traditional Chinese Medicine (temperature 21–25 °C; humidity 50–60%; well-ventilated cages) kept on 12 h/12 h light–dark cycle. Food and water were available ad libitum. The study protocol was approved by our university’s Animal Care and Use Committee. Before experimental preparation, all rats were housed for 1 week for environmental adaptation and observation.

### Preparation of Fusu agent

Fusu agent, an extract from six species of medical herbs: *Aconitum carmichaeli Debx, Carapax Testudinis, Fructus Amomi, Rhizome Zingiberis, Radix Glycyrrhizae Preparata and Herba Ephedrae*, was prepared by the Pharmacy Department of the Affiliated Hospital of Chengdu University of Traditional Chinese Medicine. Briefly, the above materials were concerted in a ratio of 6:3:4:3:2:3. First, Aconitum carmichaeli Debx and Carapax Testudinis were soaked for 30 min in water and decocted for 30 min. Then, other four species of medical herbs (Fructus Amomi, Rhizome Zingiberis, Radix Glycyrrhizae Preparata and Herba Ephedrae) were added, and decocted three times with boiling distilled water for 1 h. The decoction was filtered, collected and concentrated to some kind of evenly thick paste. The thick paste was dried in an oven for 48 h and pounded into powder. In this experiment, the animal dose of liqi (Fusu agent) was the dose of crude drug.

**Fig. 1:**
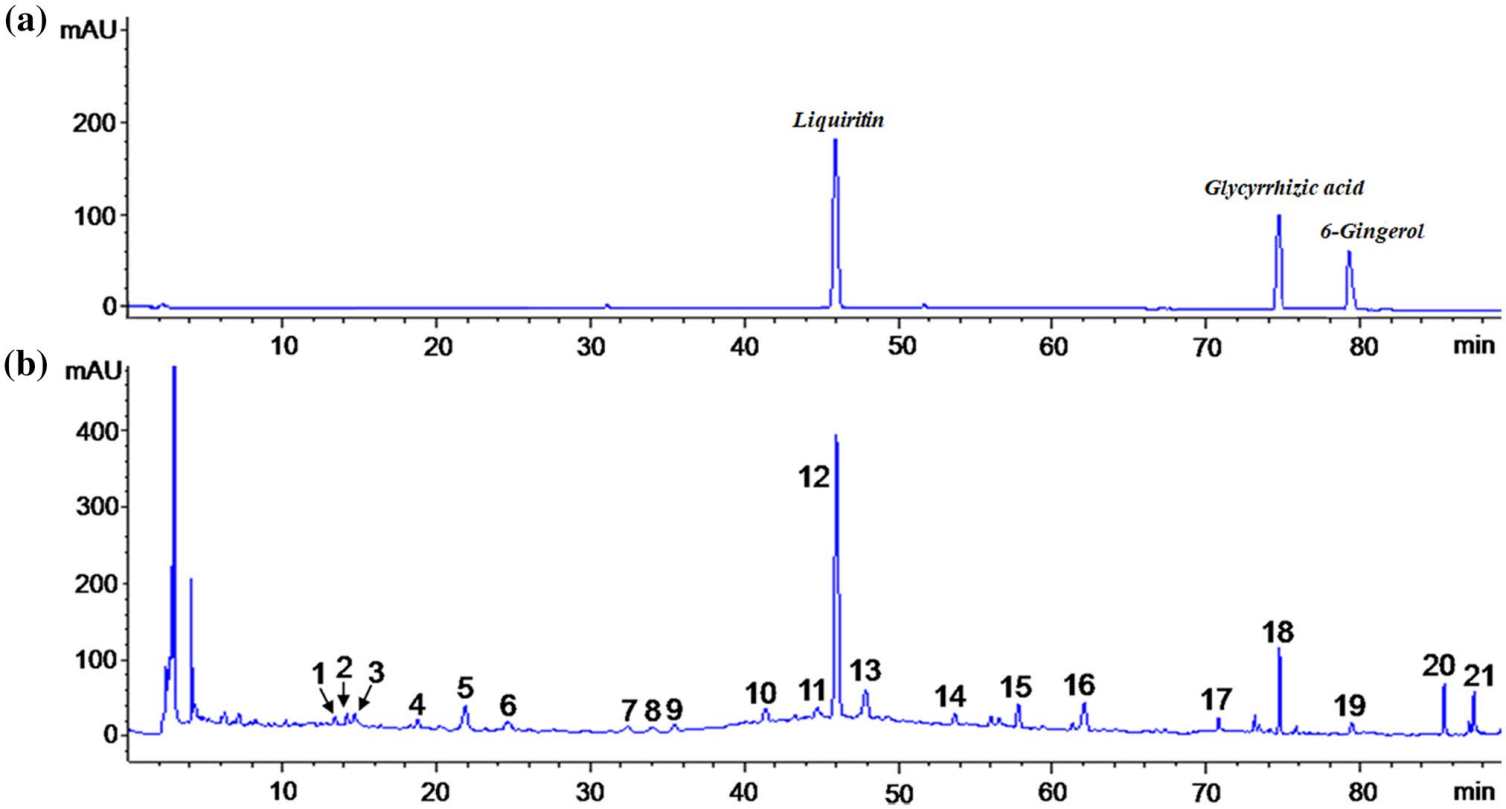
The fingerprint of the Fusu agent by HPLC. **a** The reference standard; **b** The 21 characteristic peaks of Fusu agent fingerprint. The No. 12 peak (liquiritin), No. 18 peak (glycyrrhizic acid) and No. 19 peak (6-gingerol) are determined compared with reference standard. The No. 12 peak (Liquiritin) can be used as the quality control and reference peak

The fingerprint of the Fusu agent by HPLC (Agilent Technologies 1200 Series) chromatograph was carried out to the quality control. As shown in Fig. 1, the fingerprint of the Fusu is composed of 21 characteristic peaks. The No. 12 peak, No. 18 peak and No. 19 peak are liquiritin, glycyrrhizic acid and 6-gingerol compared with reference standard, respectively. Especially, the No.12 peak (liquiritin) is used as the quality control and reference peak by its properties of good separation, no impurity interference and relatively stability.

### LPS-induced ALI model and grouping

Animals were randomly divided into five groups (*n* = 12):1. Untreated animals (normally control group, NC); 2. The LPS-induced model group (M): rats treated with an i.p. injection of 3 mg/kg of lipopolysaccharides (LPS) from Escherichia coli 0127:B8 (Sigma, L3129); 3.The high-dose Fusu agent and ALI group(HD): Fusu agent was administered intraperitoneally (10.5 g/kg) 12 h before i.p. injection of 3 mg/kg of LPS; 4. The medium-dose Fusu agent and ALI group(MD): Fusu agent was administered intraperitoneally (7.875 g/kg) 12 h before i.p. injection of 3 mg/kg of LPS; 5. The lower dose Fusu agent and ALI group (LD): Fusu agent was administered intraperitoneally (2.625 g/kg) 12 h before i.p. injection of 3 mg/kg of LPS. Moreover, the NC group and M group received a volume of saline equivalent instead of Fusu agent. The dose of Fusu agent to carry out this experiment was chosen on the basis of experimental methodology of pharmacology [20]. The six animals were killed and lungs were harvested at the 2 and 6 h after LPS challenge in all groups.

### Lung histology

After the animals were killed, left lungs were quickly removed and fixed with phosphate-buffered 4%paraformaldehyde solution (Sigma), dehydrated, and embedded in paraffin. Hematoxylin–eosin-stained lung tissue sections were evaluated for the severity of lung injury according to Smith et al [21]. Based on a scale of 0–4 (0, absent and appears normal; 1, light; 2, moderate; 3, strong; 4, intense) for edema, inflammation, hemorrhage, necrosis and hyaline membrane formation, the lung injury of three–five ×400 magnification images was graded and calculated by the mean score of the above parameters.

### Immunohistochemistry

The formalin-fixed and paraffin-embedded (FFPE) lung samples (4 µm) were performed immunohistochemistry (IHC) analysis according to the Elivision plus Polyer HRP (Mouse/Rabbit) IHC Kit protocol (MXB biotechnology, China). Briefly, tissue sections were deparaffinized using xylene and graded ethanol and were incubated for 3 h at 37 °C with primary rabbit polyclonal antibodies against CD31 (Abcam, ab28364) at a concentration of 1:40. Capillaries were identified by positive staining for CD31.To quantify CD31 expression, signal intensity analysis of three–five × 400 magnification images was performed on digitized images using the Image-Pro Plus software (Olympus, Tokyo, Japan).

### Measurement of HPA, TNF-α and IL-10 levels

The blood was obtained from the main artery of the rats in all groups at 2 and 6 h post-LPS-induced, and the plasma was isolated by centrifugation (12,000 g, 15 min) and stored at − 80 °C. The plasma levels of HPA, TNF-α and IL-10 were measured using ELISA kits (Shanghai Westang biotechnology, China) according to the manufacturer’s instructions.

### Quantitative real-time PCR

The lung obtained from the rats in all groups at 2 and 6 h post-LPS-induced was immediately put into the RNAsafety Stabilization Reagent (Shanghai Biotechnology Corporation, China) and then stored into − 80 °C until use. Total RNA was extracted and purified using RNAiso Plus Kit (TAKARA #9109), and RNA integration was inspected by Agilent Bioanalyzer 2100 (Agilent technologies, USA). Then, quantitative real-time PCR **(**QPCR**)** reaction was performed using ReverTra Ace qPCR Kit (TOYOBO #FSQ-101) and Power SYBR^®^ Green RT-PCR Reagents Kit (#4368577, ABI, USA) according to the manufacturer’s user guide. The primers were designed by Sangon Biotech. Primers included: HPA, forward primer (F), 5′-CCCTCTCTGTCCTGCTGTCTGT-3′and reversed primer(R),5′-CAGTTGCAGCCATCACATGAC-3′; SDC-1,F,5′-CCCTCTCTGTCCTGCTGTCTGT-3′ and R,5′-CAGTTGCAGCCATCACATGAC-3′; GPC-1, F, 5′-CACCACTTGGAGCCTTGTATC-3′ and R, 5′-GGAAGGGAAGATGGAGCCTTG-3′;ICAM-1, F, 5′-TCTGGGGAAAGATCATACGG-3′ and R, 5′-AGGGGTCCCAGAGAGGTCTA-3′;β-actin, F, 5′-TCTGTGTGGATTGGTGGCTCTA-3′ and R, 5′-CTGCTTGCTGATCCACATCTG-3′. QPCR was performed on ABI 7900 HT Sequence Detection System, and data were analyzed with 7900 System SDS software. β-actin was used as normalization control. Relative expression values from three independent experiments were calculated following the 2^− ΔΔCt^ method.

### Western blot analysis

The primary antibodies used were: Heparanase1/HPA1(1:2000) (Abcam, ab128931), Glypican1/GPC1 (1:1000) (Abcam, ab106003), Syndecan-1/SDC-1 (1:2000) (Abcam, ab128936), ICAM1 (1ug /ml) (Abcam, ab124760), GAPDH (1:2500) (Abcam, ab9485)—Loading Control. HRP-linked secondary antibody (#7074) was from Cell Signaling Technology, Inc.

Total protein was extracted using T-PER™ Tissue Protein Extraction Reagent (Pierce #78510), the concentration and quantitation was measured by Pierce™ BCA Protein Assays (Pierce #23227). Then, the 20 μg total protein resolved by 10% SDS-polyacrylamide gel electrophoresis was transferred to polyvinylidene difluoride (PVDF) membrane (Millipore), blocked with 5% non-fat milk and 0.1% Tween-20 for 1 h, and incubated with primary and secondary antibodies overnight at 4 °C and 1 h at room temperature, respectively. Signals were detected by exposure to X-ray films after treatment with the ECL Western Blotting Substrate kit (Pierce).

### Statistical analysis

Statistical analysis of the data was conducted using the SPSS 18 software (Chicago, IL, USA). Experimental data were expressed as the mean ± standard deviation and analyzed using two-tail Student’s *t* test. *P* < 0.05 was considered to indicate a statistically significant difference.

## Results

### Protective effect of Fusu agent on LPS-induced ALI in rats

The intratracheal administration of LPS has been widely accepted as a clinically relevant animal model of ALI/ARDS. To explore the appropriate doses of LPS, the doses of LPS (1–5 mg/kg) and time gradient (0.5, 1, 2, 3, 4, 5 h) were carried out. The pathological scores of the lung injury indicated that low and high doses of LPS can cause acute lung injury, but it takes longer for low doses (data not shown). Combined with previous experimental reports, 3 mg/kg dose of LPS administration for 2 h was considered as an induced condition of ALI.

**Fig. 2:**
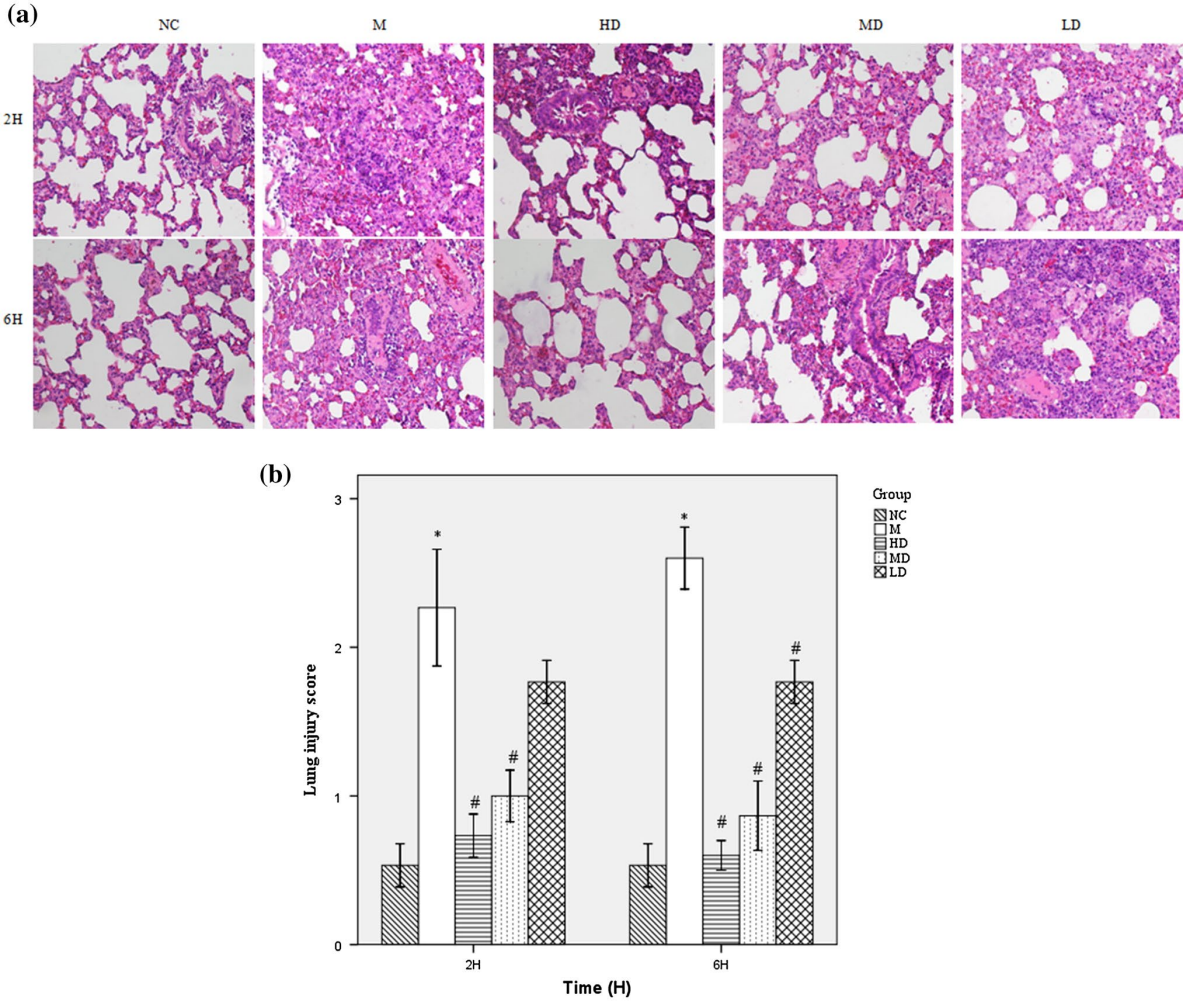
Protective effect of Fusu agent on LPS-induced ALI. **a** Representative histology sections of lung tissue at 2 and 6 h by hematoxylin and eosin staining (×200); **b** The pathological lung injury score at 2 and 6 h (*N* = 6). *P < 0.05 versus NC group, #*P* < 0.05 versus M group. (*NC* the normally control group, *M* the LPS-induced model group, *HD* the high-dose Fusu agent and ALI group, *MD* the medium-dose Fusu agent and ALI group, *LD* the lower dose Fusu agent and ALI group)

To investigate the protective effect of Fusu agent on LPS-induced ALI, Fusu agent was administered intraperitoneally (10.5, 7.875 and 2.625 g/kg) at 12 h before i.p. injection of 3 mg/kg of LPS. Then, we evaluated the severity of lung injury at 2 and 6 h after LPS administration. Compared with the control group, inflammatory cell infiltration, alveolar hemorrhage and exudation, deciduous and necrosis of type II epithelial cells, etc., were caused in LPS-induced ALI model group as previously reported. With the increasing LPS-induced time, the degree of lung injury becomes more and more serious. In contrast, administration of Fusu agent could significantly improve the lung injury (Fig. 2a).

Then, we evaluated the severity of LPS-induced lung injury according to Smith score. As shown in Fig. 2b, quantal scoring of histological lung injury in the ALI model group was significantly higher than in the control group at 2 and 6 h (*P* < 0.05 versus NC). Pre-treatment of ALI rats with Fusu agent, significantly reduced the quantitative scoring of histological lung injury (*P* < 0.05 versus M group) except the lower dose group at 2 h. Together, these data showed that early pre-treatment of Fusu agent in ALI rats has a protective effect on the LPS-induced ALI, although lung injury was not completely prevented.

### Fusu agent inhibits ESL injury

**Fig. 3:**
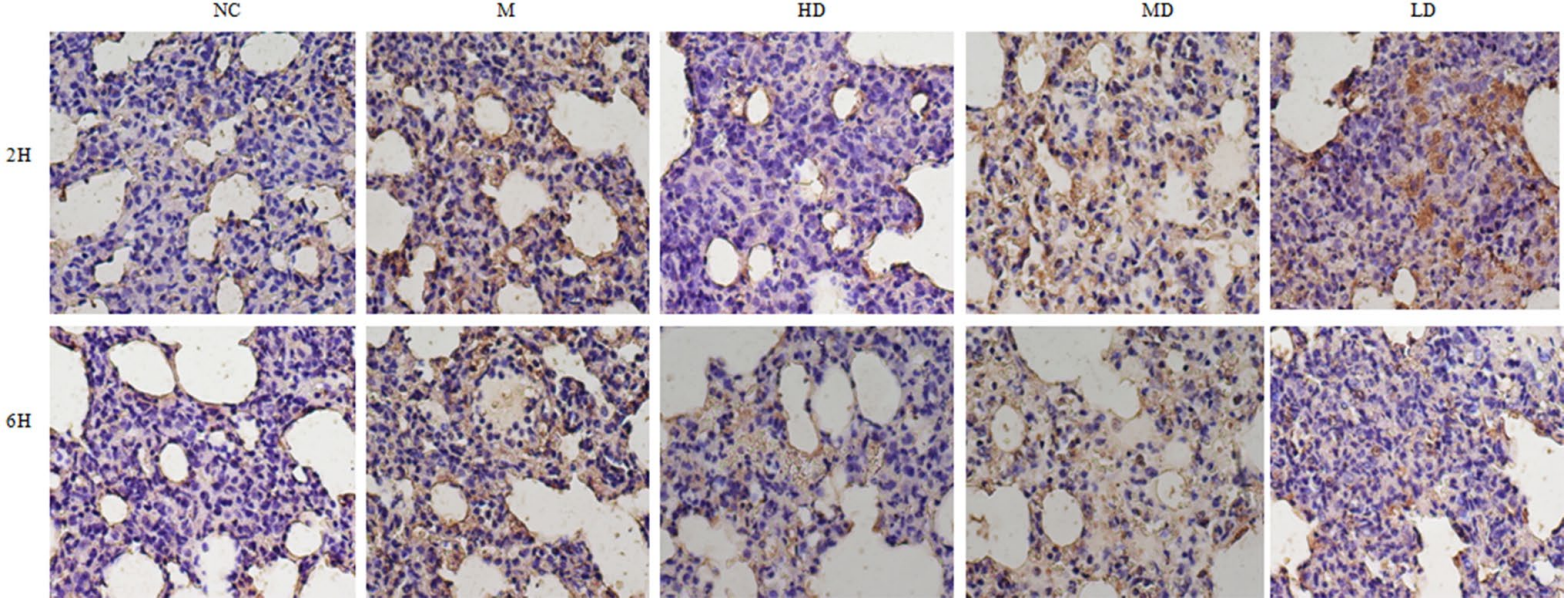
CD31 immunohistochemical staining. Representative histology sections of lung tissue at 2 and 6 h by CD31 immunohistochemical staining (×200) at 2 and 6 h (*N* = 6). CD31 is a specific marker for endothelial vascular lesions. Compared with NC group, there was positive staining signal clearly in M group. However, vascular endothelium of early pre-treatment with Fusu agent is clearer and vascular integrity is recognizable

The vascular endothelial cell and endothelial surface layer (ESL) injury is the initial event in pathophysiological events of ALI which can result in alveolo-capillary barrier leakage, as well as interstitium and alveoli edema formation finally. CD31 (endothelial cell adhesion molecule-1), which is involved in vascular endothelial cells, platelets, monocytes, etc., is a major component mediating leukocyte–endothelial adhesion. In IHC staining, CD31 is a specific marker for endothelial vascular lesions [22]. Compared with the control group, there were positive staining signal clearly in the vessel, with pulmonary endothelial loss seriously in LPS-induced ALI model group. In contrast, early pre-treatment with Fusu agent in ALI rats showed that vascular endothelium is clear and vascular integrity is recognizable (Fig. 3). These data proved that Fusu agent can reduce the vascular endothelium loss and injury.

### Effects of Fusu agent on the expression of HPA, TNF-α and IL-10

**Fig. 4:**
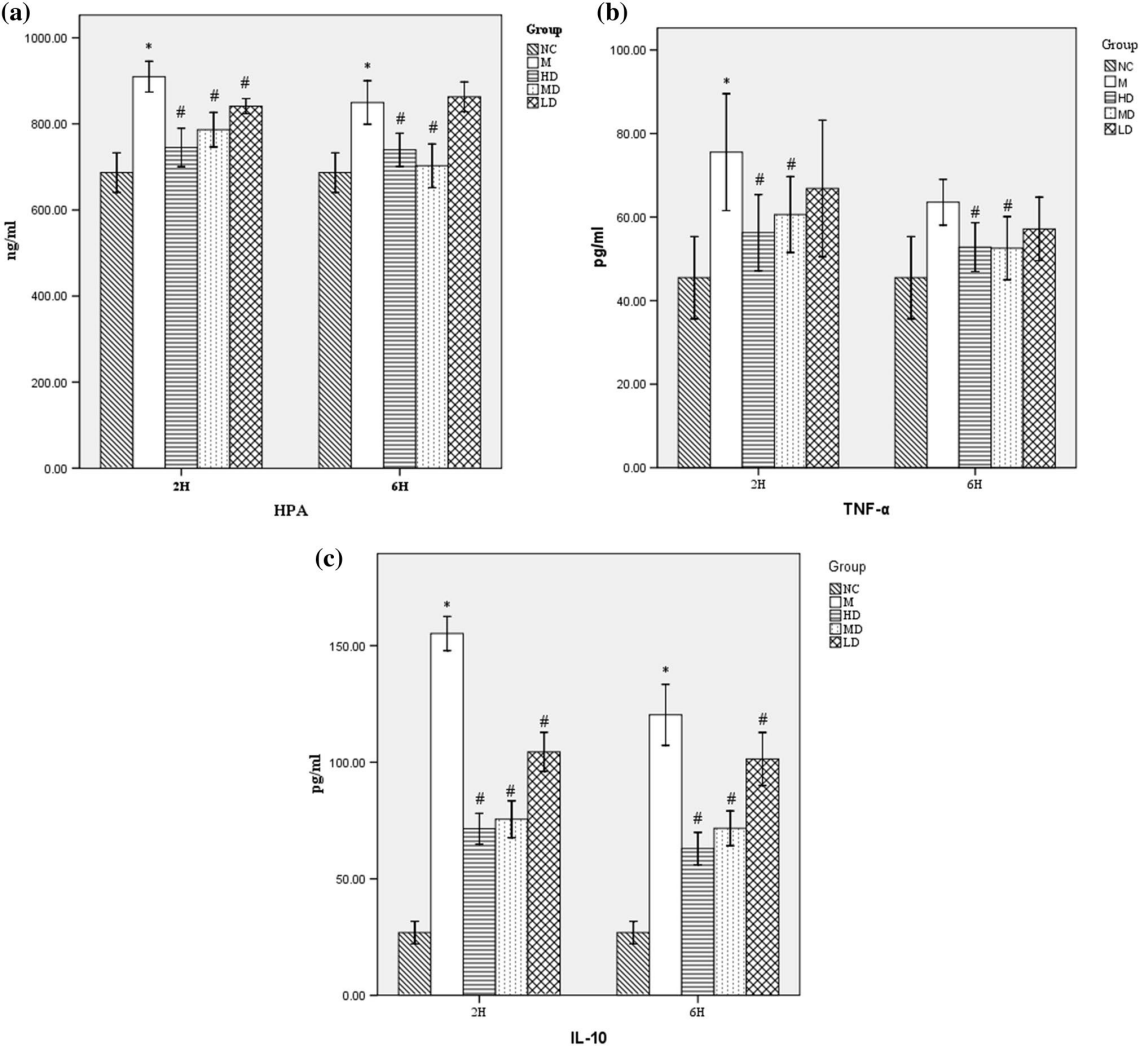
Effects of Fusu agent on the levels of HPA, TNF-α and IL-10. All data were obtained at 2 and 6 h after LPS aspiration. **a** HPA; **b** TNF-α; **c** IL-10. In M group, the expression of HPA, TNF-α and IL-10 was markedly increased after LPS stimulation. Fusu agent can significantly inhibit the expression of these cytokines, especially in HD and MD group. *N* = 6, *P < 0.05 versus NC group, #*P* < 0.05 versus M group

To demonstrate that heparanase activation is responsible for the development of ALI pathophysiology and ESL damage during LPS-induced ALI rats, we assessed the effects of calcitriol on the levels of heparanase in the plasma. As shown in Fig. 4a, LPS administration increased the expression of heparanase significantly. Conversely, early pre-treatment with Fusu agent in ALI rats can significantly inhibit heparanase expression.

TNF-α and IL-10 play an important role in the inflammatory responses. Our assay identified the effects of Fusu agent on the levels of TNF-α and IL-10 by LPS-induced inflammation response. After LPS stimulation, the results showed that TNF-α and IL-10 markedly increased. Compared with the ALI model group, Fusu agent can significantly inhibit the expression of these inflammation cytokines (Fig. 4b, c). However, no significant difference between TNF-α the lower dose group and the ALI model group was observed. It is interesting to note that the expression of these inflammation factors was higher in the 2 h after LPS stimulation than 6 h; acute inflammation as the initial event is a non-specific response to injury and damage.

### Fusu agent regulates HPA mRNA expression, but not expression of GPC-1, SDC-1 and ICAM-1 significantly

**Fig. 5:**
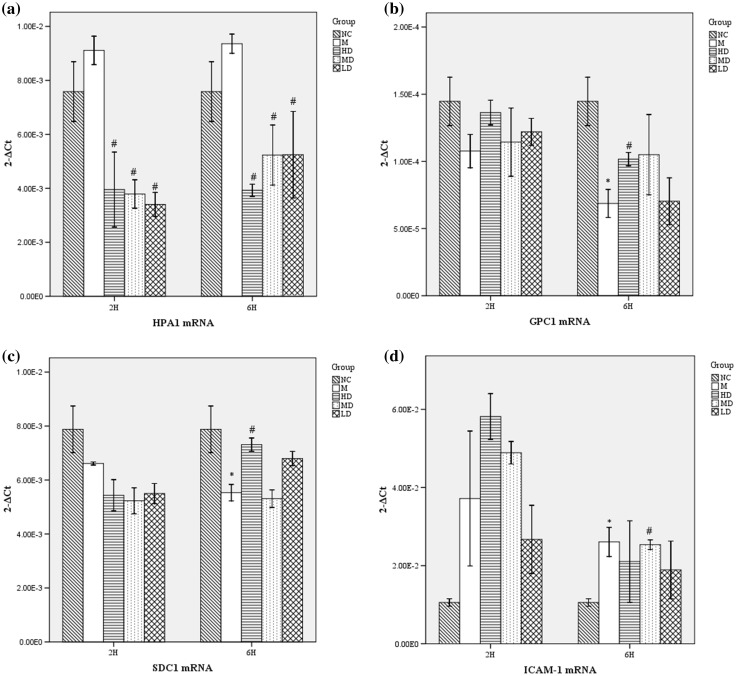
Fusu agent up-regulates HPA mRNA, but does not impact GCP1, SDC1 and ICAM1significantly. The lung tissues of 2 and 6 h after LPS aspiration were moved for QPCR analysis. Relative mRNA expression was normalized by β-actin in each sample. **a** HPA: the mRNA expression of HPA increased in M group, and Fusu agent can significantly inhibit the expression of HPA. GPC1 (**b)** and SDC1 (**c)**: The HSPGs and GPC1 mRNA expressions were down-regulated in M group, but Fusu agent showed no significant effect. **d** ICAM-1: the increased ICAM-1 mRNA was expressed compared with NC group, and Fusu agent can inhibit ICAM-1 mRNA expression at 6 and not 2 h. *N* = 6, *P < 0.05 versus NC group, #*P* < 0.05 versus M group

On the basis of the previous findings, our study also demonstrates that endothelial heparanase expression is activated constitutively, to mediate the rapid thinning of the pulmonary ESL after LPS exposure. Fig. 5a shows that mRNA expression of HPA increased after LPS-induced model group, though not significantly in the control group. Compared with the ALI model group, Fusu agent can significantly inhibit the expression of HPA mRNA.

The ESL permeability is heavily dependent on HSPGs, GPC1 and SDC1 expression is necessary for ESL mediation, as the core proteins of HSPGs. ICAM-1 exposes previously, allowing neutrophil–endothelial recognition and adhesion after ESL loss. In our study, we found that GPC1 and SDC1 mRNA (Fig. 5b, c) expressions were down-regulated after LPS-induced, as well as increased ICAM-1 mRNA expression (Fig. 5d), compared with the control group. Conversely, Fusu agent can inhibit ICAM-1 mRNA expression and increase the mRNA expression of GPC1 and SDC1.The trend is more obvious at 6 h after LPS-induced, and the high-dose Fusu agent indicates a significant effect.

### Fusu agent regulates HPA, GPC-1, SDC-1 and ICAM-1 protein significantly

**Fig. 6:**
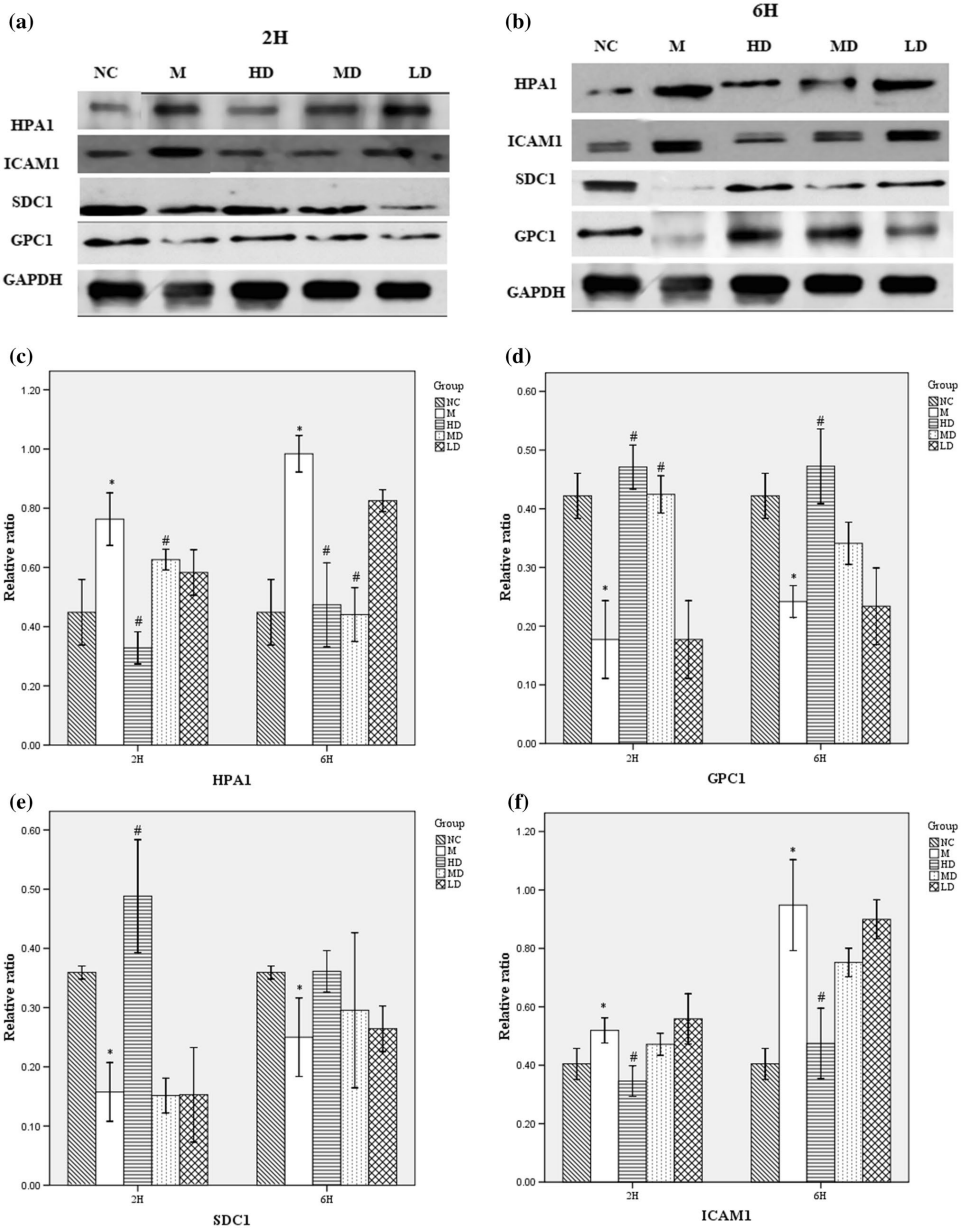
Fusu agent regulates HPA, GCP1, SDC1 and ICAM1 protein significantly. Representative immunoblot was shown 2 (**a**) and 6 h (**b**) after LPS aspiration by western blot analysis. As shown, LPS administration enhanced the increasing expression of HPA1 and ICAM1, but inhibited SDC1 and GPC1 protein expression significantly. However, Fusu agent can alter the expression of these proteins conversely. Relative protein expression was normalized by GAPDH protein in each sample, and the ratios were obtained for each group to examine the HPA (**c**), GPC1 (**d**), SDC1 (**e**) and ICAM-1 (**f**). *N* = 6, *P < 0.05 versus NC group, #*P* < 0.05 versus M group

Next, western blot was used to measure the HPA, GPC-1, SDC-1 and ICAM-1 protein expressions at 2 and 6 h after LPS treatment (Fig. 6a, b). Compared with the control group, LPS caused significant increase in HPA protein expression level. The increase in HPA protein suggests that LPS-induced ALI led to HPA activation. However, pre-treatment of ALI rats with Fusu agent resulted in significantly reduced protein expression of HPA at 2 and 6 h, regarding the lower dose group (Fig. 6c).

GPC1 and SDC1 protein (Fig. 6d, e) expressions were down-regulated, as well as increased ICAM-1 protein expression (Fig. 6f) significantly, compared with the control group at 2 and 6 h after LPS treatment. The pre-treatment of ALI model with high-dose Fusu agent can increase the protein expression of GPC1 and SDC1 significantly and reduce protein expression of ICAM-1. The medium-dose and lower dose Fusu agent can also alter the expression of these proteins, but not significantly.

## Discussion

In this study, our findings demonstrate Fusu agent can attenuate LPS-induced acute lung injury by inhibiting endothelial surface layer injury to relieve lung capillary leakage, as a traditional Chinese medicine. ALI is a complex disorder with high morbidity and mortality, resulting from vascular endothelial injury and alveolar epithelial injury [7].

Animal models provide a bridge between clinical medicine and the laboratory bench. Mechanistic studies and hypotheses can be performed by successful animal models. The LPS-induced ALI rat model was one of the earliest and most commonly used approaches, as one way for us to test the role of Fusu agent in ALI [23]. In accordance with our previous clinical and animal studies, Fusu agent can prevent the lung injury of LPS-induced ALI (Fig. 1). Further, the protective mechanisms of Fusu agent are needed to be elucidated in ALI.

The endocapillary layer might cover the entire endothelial surface, contributing to microvascular flow, plasma flow and microvascular networks [24]. As the initial event of ALI, our results demonstrate that there is rapid ESL loss and injury in LPS-induced rats. IHC staining proved that Fusu agent can inhibit ESL injury. At the same time, inflammatory cytokine’s release is the most frequent clinical feature during ALI to cause edema, reduced effective circulating volume and endothelium [25]. Previous reports demonstrated TNF-α has a key role in ALI and can damage renal endothelial cells [26, 27]. In our study, TNF-α increased significantly after LPS administration, while Fusu agent can inhibit the lever of TNF-αsignificantly. For more comprehensive results, we also measured a potent anti-inflammatory cytokine IL-10, the deficiency is thought to augment acute lung injury following hemorrhagic shock [28, 29]. In the initial time of LPS-induced ALI rats, the expression of IL-10 increased significantly for immune response, but the expression of IL-10 decreased the effect of Fusu agent versus LPS-induced ALI model to balance the immune system. These data demonstrate that Fusu agent plays important roles in balancing the immune system of whole life body.

Further, the underlying mechanisms for Fusu agent can inhibit ESL injury in LPS-induced ALI were investigated deeply. Under physiological conditions, ESL arises from the association of components of the endothelial glycocalyx layer (EGL) with blood-borne molecules [15, 30]. The proteoglycans along with their associated glycosaminoglycan (GAG) side chains are major constituents of EGL, as proteins that contain specific sites where heparan sulfate (HS) combination family—sulfated GAGs are covalently attached [31]. The interactions between GAGs and proteins are highly dependent on the conditions of their local microenvironment and impacts EGL structure [32, 33]. The glypicans and syndecans, as core proteins HS proteoglycans (HSPGs), contain mostly HS side chains. In mammalian cells, heparanases are endoglycosidases that cleave HS chains at specific intrachain sites, and heparanase-1 is primarily one single-dominant heparanase enzyme. Recent studies suggest that increased heparanase-1 is responsible for the initialed composition of internalized HSPGs, attributed to degradation of HS chains at the cell surface [12, 14, 34, 35]. In our study, ELISA, QPCR and western bolt analysis showed that heparanase expression increased significantly after LPS exposure, accompanied with loss and injury of the pulmonary ESL. GPC-1 and SDC-1, as representative core proteins HS HSPGs family, increased significantly after LPS exposure by western bolt analysis. Fusu agent can inhibit the lever of HPA mRNA and protein, GPC-1 and SDC-1 proteins significantly, especially the high-dose Fusu agent. In transcriptional level of GPC-1 and SDC-1, there is no significant effect on LPS-induced ALI after Fusu agent treatment. The recent study found that heparanase activation (with consequent HSPGs degradation) is necessary for ESL loss in the development of LPS-induced ALI [36, 37]. These findings demonstrate that Fusu agent can decrease endothelial heparanase expression to alleviate the pulmonary ESL loss and injury after LPS exposure by mediate HSPGs.

To determine whether endothelial adhesion molecule ICAM-1 participates in the LPS-induced ALI, the QCP and western bolt analysis were conducted. Consistent with the previous study, the results showed ICAM-1 increased after LPS exposure, and the high-dose Fusu agent can inhibit the protein expression of ICAM-1 significantly [38]. Combined with study results, we found that heparanase activation (with consequent HSPGs degradation) triggers the ESL loss after LPS exposure, not only expose adhesion molecules ICAM-1, but also releases inflammatory chemokine (TNF-α), facilitating neutrophil adherence and extravasation. Fusu agent can regulate the pathogenesis to treat ALI [39, 40].

In summary, we demonstrated for the first time that the molecular mechanism of the traditional Chinese medicine “Fusu agent” has a significant effect on reducing ALI. The underlying mechanism may be that Fusu agent inhibits heparanase activation and HSPGs degradation to mitigate the ESL injury in LPS-induced ALI. Furthermore, Fusu agent also has a significant inhibitory effect on TNF-α and ICAM-1, which makes a contribution to subsequent inflammatory cell influx and neutrophil adhesion in ALI. The findings shed light on the pharmacologic basis for the clinical application of traditional Chinese medicine in treating ALI.
